# Spatial and Temporal Characteristics and Drivers of Agricultural Carbon Emissions in Jiangsu Province, China

**DOI:** 10.3390/ijerph191912463

**Published:** 2022-09-30

**Authors:** Chao Hu, Jin Fan, Jian Chen

**Affiliations:** 1 College of Economics and Management, Nanjing Forestry University, Nanjing 210037, China; 2 Economic Development Quality Research Center Base, Nanjing Forestry University, Nanjing 210037, China

**Keywords:** agricultural carbon emissions, agricultural carbon emission density, spatial and temporal distribution characteristics, driving factors, global autocorrelation, STIRPAT, Jiangsu Province

## Abstract

Scientific measurement and analysis of the spatial and temporal distribution characteristics of agricultural carbon emissions (ACEs) and the influencing factors are important prerequisites for the formulation of reasonable ACEs reduction policies. Compared with previous studies, this paper fully considers the heterogeneity of rice carbon emission coefficients, measures and analyzes the spatial and temporal characteristics of ACEs in Jiangsu Province from three carbon sources, including agricultural land use, rice cultivation, and livestock and poultry breeding, and explores spatial clustering patterns and driving factors, which can provide a reference for agricultural low-carbon production. The results indicate that from 2005 to 2020, Jiangsu’s ACEs showed a decreasing trend, with an average annual decrease of 0.32%, while agricultural carbon emission density (ACED) showed an increasing trend, with an average annual increase of 0.16%. The area with the highest values for ACEs is concentrated in the northern region of Jiangsu, while the areas with the highest values for ACED are distributed in the southern region. The spatial clustering characteristics of ACEs have been strengthening. The “H-H” agglomeration is mainly concentrated in Lianyungang and Suqian, while the “L-L” agglomeration is concentrated in Zhenjiang, Changzhou, and Wuxi. Each 1% change in rural population, economic development level, agricultural technology factors, agricultural industry structure, urbanization level, rural investment, and per capita disposable income of farmers causes changes of 0.112%, −0.127%, −0.116%, 0.192%, −0.110%, −0.114%, and −0.123% in Jiangsu’s ACEs, respectively. To promote carbon emission reduction in agriculture in Jiangsu Province, we should actively promote the development of regional synergistic carbon reduction, accelerate the construction of new urbanization, and guide the coordinated development of agriculture, forestry, animal husbandry, and fisheries industries.

## 1. Introduction

Climate change is closely related to human activities. Since the industrial revolution, the excessive use of fossil fuels such as coal, oil, and natural gas by humans and the emission of large amounts of greenhouse gases are the main causes of global climate change, and large-scale deforestation and grassland destruction have exacerbated this process [1,2]. The agricultural sector is receiving more and more attention because it plays an important role in reducing greenhouse gases due to its dual attributes as a carbon sink and a carbon source. Studies have shown that the total carbon emissions caused by agricultural production activities reach 15% of the total carbon emissions, and the carbon emissions from agriculture should not be underestimated [3]. As a large agricultural producing country, China has more carbon emissions caused by agricultural production, which accounts for about 17% of the total carbon emissions and is growing at a rate of 1.67% per year [4]. Encouragingly, the Chinese government has pledged to bring CO_2_ emissions down in absolute terms by 2030, with carbon emissions intensity reduced to 50–60% of 2005 levels [5]. Therefore, China urgently needs to reduce ACEs by developing low-carbon agriculture. Because controlling carbon emissions in agricultural production is a key element in the development of low-carbon agriculture [6], strengthening research on ACEs will help to promote the goal of “zero carbon” in agricultural production and achieve the national goal of energy saving and emission reduction [7].

In recent years, there has been a gradual enrichment of research on various aspects of ACEs. Based on the research concerns, these studies can be broadly classified into the following three categories. The first category is a study on the measurement of ACEs and their spatial and temporal distribution characteristics. The selection and identification of agricultural carbon sources are crucial, which is the basis for ACEs studies. Early scholars mainly focused on farmland CO_2_ emissions, arguing that tillage leads to soil organic carbon loss and conservation tillage helps soil carbon sequestration [8,9,10]; Later, as the research progressed, chemical fertilizers and pesticides, agricultural films, energy consumption, rice cultivation, and livestock breeding also entered the research horizon of scholars, and a more comprehensive ACEs measurement index system was formed on this basis, mainly involving three different dimensions, such as agricultural material utilization, rice cultivation and livestock breeding [11,12,13]. Liu et al. (2021) [14] and Huang et al. (2022) [15] have successively measured and analyzed the performance of China’s ACEs effectively around the country. A comprehensive study shows that the growth trend of total ACEs in China is relatively obvious and has gradually stabilized or even slightly decreased in recent years, while the intensity of ACEs is in a continuous decline [16], forming a distribution pattern of Shanxi–Shandong high–high concentration area and Yunnan–Guangxi low–low concentration area [17].

The second category is the study of ACEs from a specific perspective. Some scholars have discussed the relationship between ACEs and economic growth. From the EKC perspective, economic development is the key driver for the continuous growth of ACEs in the short term, and there is an inflection point between the two in the long term, in line with the EKC characteristics [18]. From the decoupling perspective, a strong decoupling relationship between economic growth and ACEs is the best, and a strong negative decoupling state is the worst [19]. Other scholars have studied the effects of land use practices on agroecosystems at different levels and found that different farmland management practices affect soil organic carbon, changes in farmland operation scale have a threshold effect on ACEs [20,21], and free drainage is more conducive to reducing greenhouse gas emissions than controlled drainage [22]. A large number of scholars have conducted relevant studies in the areas of low-carbon behavior of farmers [23], the agricultural carbon footprint [24], implied carbon emissions from agricultural trade [25], ACEs efficiency [26], and ACEs reduction potential [27] and have come up with a series of enlightening conclusions.

The third category is studies related to the drivers of ACEs. The identification of key ACEs drivers helps to scientifically guide the process of carbon emission reduction and is one of the hot topics in existing ACEs research. The representative research methods are factor decomposition methods, including the IDA model, Kaya formula, LMDI decomposition method, IPAT model, STIRPAT model and other models [28]. In addition, some scholars have used random forest models to simulate ACEs scenarios [29,30,31]. Marçal et al. (2021) used a random forest model to assess and predict carbon stocks in rangelands and forests [32]. Li et al. (2016) analyzed the key factors of energy GHG emissions from the agricultural sector in European countries using the IDA model and found that rising energy intensity was the main reason for the increase in CO_2_ [33]. Applying the Kaya equation, Okorie et al. (2022) found a decrease in the contribution of agricultural value added to the total level of ACEs in Nigeria [34]. Li et al. (2014) applied the extended Kaya equation to find that implementing agricultural subsidy policies can help reduce CO_2_ emissions [35]. Akram1 et al. (2019) applied the LMDI model and found that the energy mix effect is the largest driver of CO_2_ emissions in Pakistan [36]. Huang et al. (2022) applied the LMDI model to analyze the main drivers of the carbon footprint of cotton in China [37]. Tian et al. (2016) used the IPAT model to analyze the factors influencing the changes in the water footprint of five major food crops in China [38]. These decomposition methods decompose ACEs into specific factors such as agricultural economic growth, agricultural production efficiency and agricultural population size, and assign them economic meanings. However, the limited decomposition factors are susceptible to challenge as carbon emissions are influenced by multiple factors [39]. The STIRPAT model can overcome these shortcomings and thus has become a common method for identifying the drivers of ACEs [40]. Compared with other decomposition methods, the STIRPAT model can be extended according to the actual situation of the study area, which makes the realistic basis and model construction more consistent and feasible, and thus more widely used in the study of environment-related problems [41].

In summary, the existing research results on ACEs are rich, roughly covering carbon emission measurement, exploration of influencing factors, and spatial and temporal distribution characteristics, but the research on the influencing factors on ACEs has, still, not yet formed a unified conclusion, and has the value of further research.

**Figure A1:**
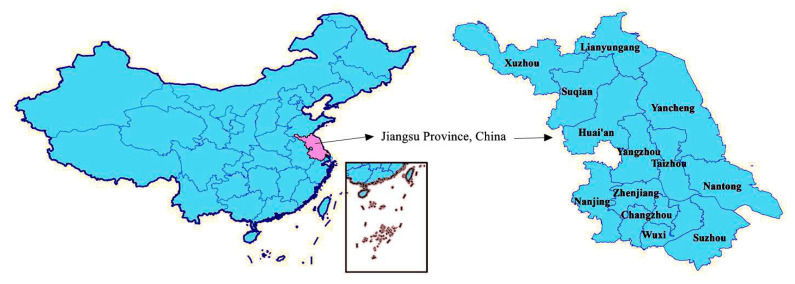
Geographical location and administrative division map of Jiangsu Province.

Jiangsu Province is a large agricultural province in China, with excellent geographical and climatic conditions, and is an important grain province in the middle and lower reaches of the Yangtze River (Figure A1) [42]. The topography of Jiangsu Province is dominated by plains, which account for 86.89% of the area. Jiangsu Province is not only a developed economic province in China but also has a leading level of agricultural development in the country. In 2020, the total agricultural output value for Jiangsu Province reached USD 112.36 billion, accounting for 5.72% of the country’s agricultural output; grain production reached 37.29 million tons, the seventh highest-producing place in the country. While the economic level is increasing, the pressure on resources and the environment is also rising significantly [43]. Jiangsu Province applied 2.875 million tons of fertilizer in 2020, accounting for 5.35% of the country’s fertilizer usage. Jiangsu Province is promoting the green development of agriculture and actively creating a national agricultural green development pioneer area. Therefore, it is important to analyze the characteristics of the spatial and temporal distribution of ACEs in Jiangsu Province and research the influencing factors on its ACEs.

In this paper, we deepen the existing research in three aspects. First, due to the regional heterogeneity, the carbon emission coefficients of rice vary in different regions, and we selected the rice carbon emission coefficients with characteristics of Jiangsu Province instead of the common ones, which makes the measured results more accurate. Second, we combined the factor analysis method and STIRPAT model to solve the problem of multiple covariances of the STIRPAT model, which provides a new way of thinking to explore the influencing factors on ACEs. Third, using the latest statistical data to research the ACEs in Jiangsu Province, we made it possible to enrich previous agricultural research.

## 2. Materials and Methods

### 2.1. Data Sources and Description

In this paper, considering that Jiangsu Province is an important agricultural province in China, 2005 is the transition year between the 10th and 11th Five-Year Plans of China, and 2020 is the closing year of the 11th Five-Year Plan. The study area is set as thirteen cities in Jiangsu Province, and the period is set as 2005–2020. The data used in the study were obtained from the statistical yearbooks (2006–2021) and statistical bulletins (2006–2021) of 13 cities, as well as the Jiangsu Statistical Yearbook (2006–2021) and the China Rural Statistical Yearbook (2006–2021).

In the process of measuring ACEs in each city, all data sources were examined, and the necessary checks and adjustments were made by comparing the same types of data reported from different sources. For example, to examine the data on fertilizer application in agricultural land use, we first calculated and aggregated the fertilizer application amounts for each city in Jiangsu Province. Then, the aggregated data were compared with the province-wide values to see if the error values were acceptable. If an error was found to be too large during the inspection of these data, we adjusted it according to the relative proportion of the area sown to crops in the region. Descriptive statistical analysis of the main variables is shown in Table 1

### 2.2. Analytical Framework

**Figure 1:**
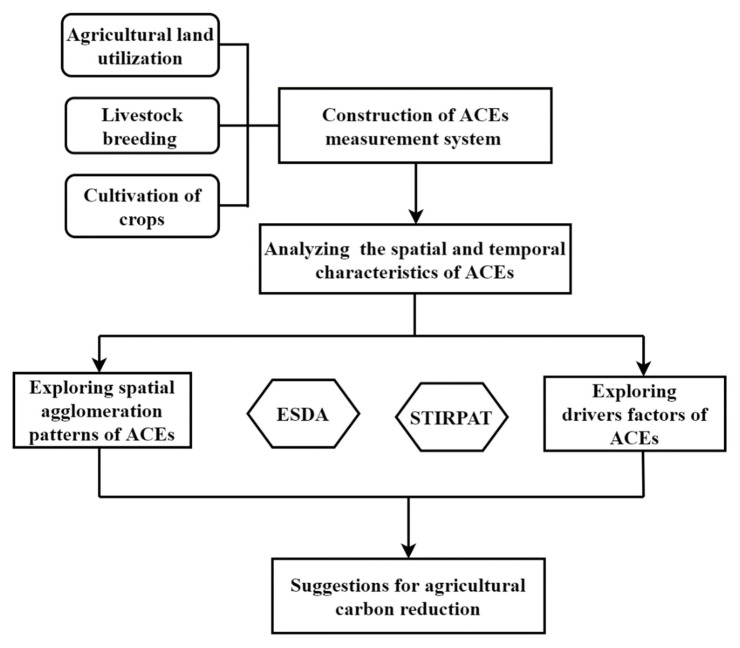
Analysis Framework.

There are several steps involved in estimating the spatial and temporal distribution characteristics and influencing factors on carbon emissions from agricultural production in Jiangsu Province (Figure 1).

### 2.3. ACEs Measurement Method

ACEs are the direct or indirect greenhouse gas emissions from the use of fertilizers, pesticides, fossil fuels, and livestock and poultry breeding processes in agricultural production. ACEs gases are mainly composed of CO_2_, CH_4,_ and N_2_O [44]. To facilitate the calculation and understanding, these three gases were converted into standard carbon for the study. The source channels of ACEs showed diversified and complex characteristics. The main sources include three aspects: firstly, carbon emissions resulting from the cultivation of agricultural lands, such as the use of fertilizers, pesticides, and other agricultural materials in the production process [45]; the second was derived from rice cultivation, which is accompanied by the production of large amounts of CH_4_ during rice production [46]; and the third one originated from animal husbandry, mainly CH_4_ and N_2_O emissions caused by animal intestinal peristalsis and improper animal manure disposal [47]. Runoff and leaching were not included because they involve cross-regional issues, and it is not easy to divide the responsibility of carbon emission attribution [48]. Jiangsu Province is located in the southeast of China, where rice is the main crop, and we considered all three sources of carbon emissions.

Drawing on the results of natural science research, and referring to the measurement methods of IPCC, Oak Ridge National Laboratory and provincial greenhouse gas emission inventories [49], the formula for ACEs is as follows:

(1) $$CE=\overset{3}{\underset{i=1}{\sum }}C_{i}=\sum^{}e_{i}\cdot \delta_{i}\;\;\;\;\;$$

In Equation (1): *CE* is the total ACEs, $C_{i}$ indicates the total carbon emission from different types of carbon sources, *i* = 1 indicates the carbon emission from agricultural land use; *i* = 2 represents the carbon emission from rice cultivation and *i* = 3 represents the carbon emission from livestock and poultry breeding. ACEs sources and corresponding carbon emission coefficients are shown in Table 2.

*ACED* refers to the carbon emissions generated per unit of crop sown area. The calculation is as follows:

(2) $$\;ACED=\frac{CE}{SA}\;$$

where *ACED* (t/km^2^) is used as one of the indicators to measure the level of ACE and *SA* denotes crop sown area.

### 2.4. Spatial Autocorrelation Evaluation Model

Spatial autocorrelation is the correlation of the same variable at different spatial locations. Many geographical phenomena, influenced by spatial processes that are continuous in their geographical distribution, are spatially autocorrelated. The exploratory spatial data analysis method includes global correlation and local correlation analysis, using the global $Moran^{\prime }s\;I$ index and the local $Moran^{\prime }s\;I$ index as statistical indicators of both, respectively [53].

The global spatial autocorrelation method analyzes the association characteristics and the degree of difference in the same variable between different observations in space by calculating the spatial autocorrelation index [54]. The specific formula is as follows:

(3) $$Global\;Moran’s\;I=\frac{\sum_{i=1}^{n}\sum_{j=1}^{n}W_{ij}\left(x_{i}-\bar{x}\right)(x_{j}-\bar{x})}{S^{2}\sum_{i=1}^{n}\sum_{j=1}^{n}W_{ij}}\;$$

(4) $$S^{2}=\overset{n}{\underset{i=1}{\sum }}\left(x_{i}-\bar{x}\right)/n\bar{x}=\overset{n}{\underset{i=1}{\sum }}x_{i}/n\;\;\;\;$$

where $x_{i}$ and $x_{j}$ represent the total ACEs (ACED) of each city in Jiangsu Province, respectively, and n is the number of observation units, i.e., 13 prefecture-level cities in Jiangsu Province; $w_{ij}$ is the spatial weight matrix using Rook adjacency, when region i and region j have a common edge, $w_{ij}=1$ and vice versa $w_{ij}=0$. The *global Moran’s I* index takes the value of [−1, 1] and its value if it lies in [−1, 0) indicates that there is a positive agglomeration characteristic of total regional agricultural carbon emission (density), and vice versa, there is a negative agglomeration characteristic; its value if 0 indicates that there is no spatial relationship between neighboring units.

Local spatial autocorrelation can explore whether there are similar or dissimilar indicator values clustered together in a local area [55]. It is calculated as follows:

(5) $$Local\;Moran’s\;I=\frac{\left(x_{i}-\bar{x}\right)\sum_{j=1}^{n}W_{ij}\left(x_{j}-\bar{x}\right)}{S^{2}}\;\;\;\;$$

A positive value for the *local Moran’s I index* indicates the existence of high–high (H-H) or low–low (L-L) spatial agglomerations in the local area, while a negative value indicates the existence of high–low (H-L) or low–high (L-H) spatial agglomerations in the local area. The larger the absolute value of the local *Moran’s I index*, the higher the degree of spatial agglomeration.

### 2.5. STIRPAT Model

The STIRPAT model was developed from the IPAT model, which overcomes the quantitative limitations of the IPAT model and avoids the shortcomings of the IPAT model that require the factors to vary in the same proportions [56], and is, therefore, widely used in empirical studies for the analysis of carbon emission drivers [57]. Its standard form is [58]:

(6) $$I=aP^{b}A^{c}T^{d}e\;\;\;\;$$

This model reveals the relationship between environmental stress (*I*), population size (*P*), economic affluence (*A*), and technology level (*T*). The ecological elasticity coefficient of each driver to environmental stress is obtained by taking the natural logarithm for both sides of the model:

(7) InI=Ina+bInP+cInA+dInT+Ine     

where *b*, *c*, and *d* are the regression coefficients of the corresponding explanatory variables, reflecting the effect of changes in each driver on environmental stress. Specifically, each 1% change in *P*, *A*, and *T* will cause b%, c%, and d% change in *I*, respectively, with the other coefficients held constant.

To further study the effects of the rural population, the level of economic development, the level of agricultural technology, the structure of the agricultural industry, the urbanization, the investment in rural fixed assets, and the per capita disposable income of rural residents in Jiangsu Province on its ACEs, the model is extended by taking into account the actual situation of its agricultural development, and the following model is constructed:

(8) $$CE=a\times P^{b}\times A^{c}\times T^{d}\times V^{e}\times U^{f}\times C^{g}\times R^{h}\times e\;\;$$

Taking the logarithm of both sides of the equation yields:

(9) InCE=Ina+bInP+cInA+dInT+eInV+fInU+gInC+hInR+Ine   

In Equation (9): *CE* is the carbon emission from agricultural production activities in Jiangsu Province (10^4^ t); P is the rural population (10^4^ people); *A* is the affluence, expressed as GDP per farmer (yuan/person); *T* is the technical factor, representing the level of agricultural technology development, expressed in terms of total agricultural machinery power (10^4^ kW); *V* is the structure of the agricultural industry, expressed as the sum of the total output value for farming and livestock as a share of agricultural output value (%); *U* is the urbanization rate, expressed in terms of the ratio of urban population to total population in Jiangsu (%); *C* is the capital factor, expressed in terms of total social fixed asset investment in agriculture, forestry, animal husbandry, and fishery industries (10^4^ yuan); *R* is the social concern factor, expressed in terms of per capita disposable income of rural residents (yuan/person). The values for *b*, *c*, *d*, e, *f*, *g*, and h are elasticity coefficients, indicating that when each 1% change in *P*, *A*, *T*, *V*, *U*, *C*, and R will cause a change in *b*%, *c*%, *d*%, *e*%, *f*%, *g*%, and *h*% of ACEs.

This paper explores the factors influencing agricultural carbon emissions in Jiangsu Province in terms of the rural population, the size of the agricultural economy, the level of agricultural technology development, and the urbanization rate, and the reasons for selecting these variables are as follows:

(1) The rural population (*P*). The rural population is an important factor influencing agricultural carbon emissions, and research basically confirms that there is a positive relationship between the two. The more people employed in agriculture, the greater the agricultural carbon emission, and the opposite is true [59].
(2) Agricultural economic factors (*A*). According to the theory of environmental Kuznets curve, there is an inverted U-shaped relationship between agricultural economic development and carbon emission, i.e., the phenomenon of polluting first and then controlling later. Agricultural economic development is the main factor driving carbon emissions, but whether it will drive the growth of carbon emissions depends on the quality and stage of economic development [60].
(3) Agricultural technology factor (*T*). Advances in agricultural technology can improve the efficiency of machinery use, which will produce fewer carbon emissions at the same level of output, expressed in terms of total agricultural machinery power [61].
(4) Agricultural industry structure (*V*). The industrial structure within the agricultural sector has a direct relationship with carbon emissions. Compared with forestry and fishery industries, plantation and livestock contribute the major share of carbon emissions [62].
(5) Urbanization rate (*U*). Urbanization reduces the rural labor force, which will prompt agricultural production agents to pay attention to scale and intensive operation, which is conducive to saving resources, improving labor productivity, and reducing agricultural carbon emissions. Therefore, we used the urbanization rate as a control variable, which is measured by the proportion of urban population to the total population [63].
(6) Capital factor (*C*). Public investment in agriculture has a small inhibitory effect on agricultural carbon emissions, but whether its inhibitory effect continues to exist as agriculture continues to develop and as the external environment changes still needs to be explored. Therefore, public investment in agriculture was used as a control variable, specifically expressed as the amount of social fixed asset investment in agriculture, forestry, animal husbandry, and fishery industries [64].
(7) Social concern factors (*R*). Higher income will enhance farmers’ sustainable development concept and promote their demand for rural environmental quality, and these factors are conducive to reducing agricultural carbon emissions, thus we included rural residents’ income as a control variable as well [65].

## 3. Results

### 3.1. Analysis of ACEs

#### 3.1.1. Analysis of the Time Series Characteristics of ACEs

**Figure 2:**
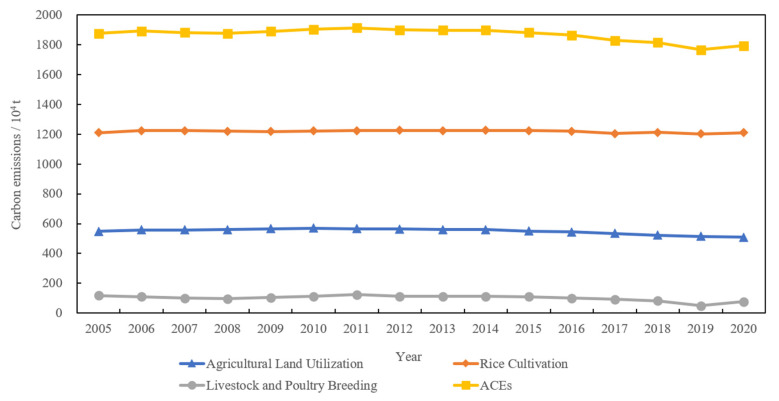
Trends of ACEs in Jiangsu Province, China, 2005–2020.

**Figure 3:**
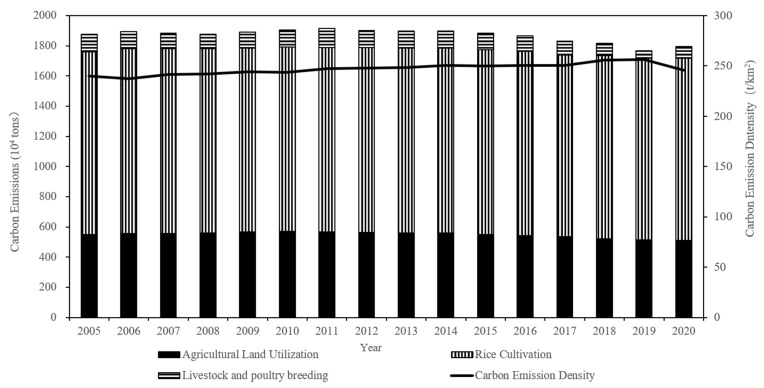
Change in ACEs and ACED in Jiangsu Province, China: 2005–2020.

According to Equation (1), the ACEs in Jiangsu Province from 2005 to 2020 were measured (Table 3, Figure 2, and Figure 3). The results show that from 2005 to 2020, the total ACEs in Jiangsu Province are in a general downward trend, decreasing from 1877.57 × 10^4^ tons in 2005 to 1795.24 × 10^4^ tons in 2020, with an average annual decrease of 0.30%. The overall ACED is in an increasing trend, increasing from 240.06 t/km^2^ in 2005 to 245.72 t/km^2^ in 2020, with an average annual growth rate of 0.16%. In terms of carbon emission sources, the proportion of the three major ACEs sources does not change much, and rice cultivation is the most important source of ACEs, accounting for more than 60% of ACEs. Agricultural land use is the second most important source of carbon emissions, contributing around 30% of carbon emissions. Livestock and poultry breeding accounts for the smallest share, with a contribution of no more than 5%. Research shows that the emissions from rice cultivation far exceed the sum of the other two types of carbon sources, and the proportion is gradually increasing. Therefore, to control ACEs, we should optimize various proportional relationships within agriculture, appropriately reduce the proportion of cultivation and expand the proportion of livestock under the premise of ensuring the safety of grain production.

From the interannual variation in ACEs, the growth rate of ACEs first increases, then decreases, and finally turns negative; therefore, the ACEs in Jiangsu during the period can be divided into three stages: rapid growth, slow decline, and accelerated reduction. The first stage is the rapid growth period (2005–2011), with a high ringgit growth rate and an average annual increment of 0.32%. This is mainly due to the comprehensive reduction in agricultural taxes and fees in China since 2004, which has greatly stimulated the enthusiasm of farmers in agricultural production and the significant growth of various agricultural production materials inputs, driving the rapid growth of ACEs. The second stage is the slow decline period (2012–2014), in which the total ACEs began to decrease slowly. The reason for this analysis is that the level of agricultural mechanization in Jiangsu Province has improved significantly. According to statistics, the comprehensive mechanization level of agriculture in Jiangsu Province was as high as 72.56% in 2012. At the same time, since 2012, the Jiangsu government has increased the control of diesel-type machinery and vigorously promoted clean energy to replace non-renewable energy, so agricultural carbon emissions began to slowly decrease. The third phase is the accelerated reduction (2015–2020), with an average annual decrease of 1.19%. This is because China began to implement the development strategy for green agricultural transformation in 2015 and has promulgated a series of low-carbon agricultural policies such as “weight loss and drug reduction”. Under the leadership of green and low-carbon policies, Jiangsu Province has promoted the construction of high-standard farmland, continued to promote pesticide and chemical fertilizer reduction and efficiency actions, and significantly improved the resource utilization of livestock and poultry manure [66]. Farmers have started to transform from traditional agricultural production methods to green and low-carbon methods and carry out low-carbon production or adopt low-carbon agricultural technologies, thus accelerating the reduction in ACEs.

From 2005 to 2020, the average annual decrease in ACEs in Jiangsu Province is −0.30%, the average annual increase in ACED is 0.16%, and the average annual decrease in crop sown area is −0.14%. In other words, the rate of decline in ACEs is higher than the rate of decline in crop sown area, and the ACED shows an upward trend, which indicates that Jiangsu Province has achieved certain results in ACEs reduction, but it does not mean that Jiangsu Province has fully realized the low-carbon development of agriculture. Jiangsu Province has only 0.06 hectares of arable land per capita, which is 59% of China’s average, but it has a population density of 742 people per square kilometer, which is 5.5 times the national average, and faces increasingly tight ecological constraints in the future. In order to realize the low-carbon development of agriculture, Jiangsu Province still needs to continue to reduce ACEs, promote the integration of agriculture to two or three industries, enhance the green production yield of agriculture, apply and promote efficient and environmentally friendly agricultural green production technology, and reduce the ACED.

#### 3.1.2. Analysis of the Spatial Characteristics of ACEs

**Figure 4:**
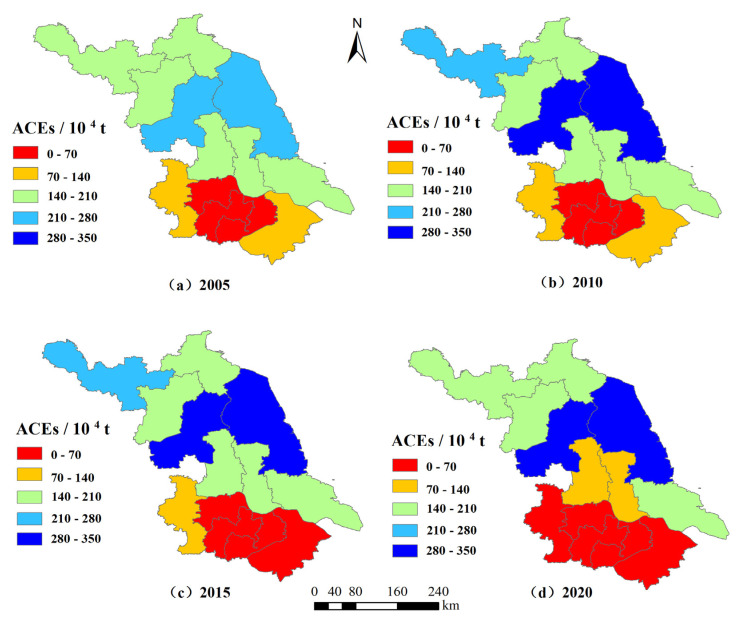
Evolution of spatial pattern of ACEs in Jiangsu Province, China.

**Figure 5:**
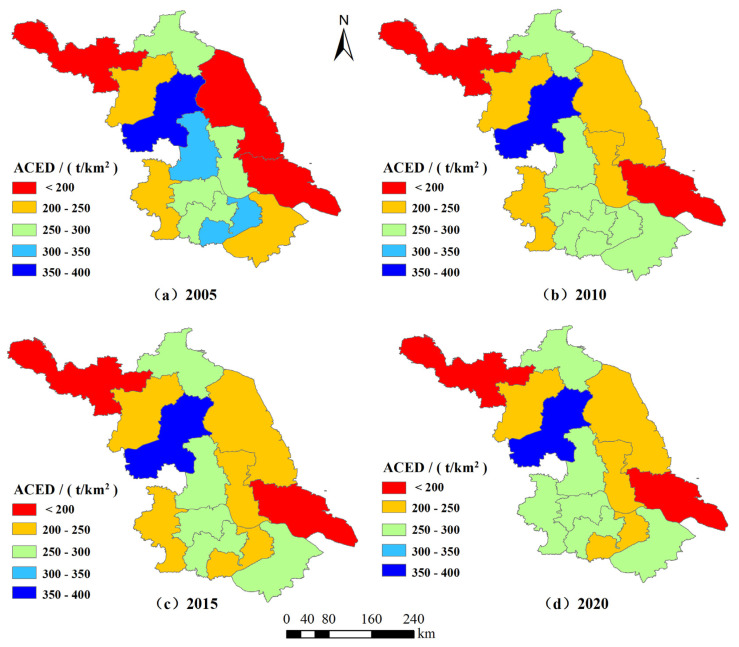
Evolution of spatial pattern of ACED in Jiangsu Province, China.

The actual values for ACEs and ACED in Jiangsu Province at the four-time points 2005, 2010, 2015, and 2020 were classified into five classes using the natural interruption point method (Table 4 and Figure 4 and Figure 5) to reveal the characteristics of municipal changes in ACEs.

According to the calculation results, the ACEs from 13 cities in Jiangsu Province vary significantly. The top five areas in terms of carbon emissions are Yancheng (307.06 × 10^4^ tons), Huai’an (299.56 × 10^4^ tons), Xuzhou (189.85 × 10^4^ tons), Suqian (174.94 × 10^4^ tons), and Lianyungang (166.69 × 10^4^ tons) and the sum of the ACEs from these five areas amounts to 1138.08 × 10^4^ tons, accounting for 63.39% of the province’s ACEs. The cities with the lowest ACEs are Wuxi (31.52 × 10^4^ tons), Changzhou (42.45 × 10^4^ tons), Zhenjiang (49.55 × 10^4^ tons), Suzhou (54.93 × 10^4^ tons), and Nanjing (66.41 × 10^4^ tons), in that order, and the sum of ACEs from these five areas is 244.87 × 104 tons, accounting for only 13.64% of the province’s total. It can be seen that the ACEs from the top five cities are equivalent to nearly five times that of the bottom five cities, indicating that there are large regional differences in the total ACEs in Jiangsu Province.

From the figure showing the regional distribution of ACEs, there is a trend of high in the north and low in the south, which is more consistent with the distribution of the agricultural economy scale in Jiangsu Province. The highest values for ACEs are concentrated in the north of Jiangsu Province, and the specific cities are Huai’an, Yancheng, Lianyungang, Suqian, and Xuzhou. These cities are also the concentration areas for agricultural production in Jiangsu Province, and their planting areas are much bigger than other regions on both an absolute and a relative scale, and the inputs of production materials such as pesticides and chemical fertilizers are relatively high, which in turn leads to higher total ACEs than other cities. In 2020, the total agricultural output value of these five cities was USD 62.45 billion, accounting for 55.9% of the province’s output. The crop area reached 4758.77 thousand hectares, accounting for 63.63% of the province’s cropping area; ACEs were 11.38 million tons, accounting for 63.40% of the province’s ACEs; and the amount of fertilizer applied was 2.02 million tons, accounting for 71.94% of the province’s fertilizer usage. The low-carbon emission area mainly includes two industrial cities, Wuxi and Changzhou, which have a relatively weak level of agricultural development due to the constraints in natural resource base conditions and other factors, so the ACEs are low. Wuxi and Changzhou’s crop sowing area is only 1.74% and 2.21% of the province respectively, ranking 13th and 12th, but their industrial revenue above the scale accounts for 15.01% and 9.41% of the province, ranking 2nd and 4th.

In terms of the growth rate of regional ACEs, there are differences in the growth rate of ACEs in different cities. Among them, Yancheng has the fastest growth rate of ACEs, from 270.85 × 10^4^ tons in 2005 to 307.06 × 10^4^ tons in 2020, an increase of 13.37%. This is followed by Huai’an, with a growth rate from 266.37 × 10^4^ tons in 2005 to 369.92 × 10^4^ tons in 2020, an increase of 12.46%. Wuxi’s ACEs have decreased to 31.52 × 10^4^ tons in 2020 from 63.46 × 10^4^ tons in 2005, which is related to the rapid development of industry in the region and the decline of the area under crop cultivation.

From Figure 5 and Table 4, Huai’an, which is in the first place, has 2.30 times the ACED of Xuzhou, which is in the last place, so it can be seen that there is a big difference in ACED among cities. In terms of regional distribution, the area with a high value for ACED is mainly represented by Huai’an City, with a value above 350 t/km^2^. The cities in the middle-value zone include Nanjing, Changzhou, Suzhou, Zhenjiang, Yangzhou, and Lianyungang, whose ACED is between 250 and 300 t/km^2^. The low-value ACED area is mainly located in Xuzhou, where heavy industry is developed, and Yancheng, where transportation is conveniently located, and its value is less than 200 t/km^2^. In terms of the ACED at different time points, except for Nanjing, Suzhou, Huai’an, and Yancheng, the densities of ACEs in other cities have been decreasing year by year. The three cities with the largest declines were Wuxi (−28.43%), Xuzhou (−19.50%), and Changzhou (−9.91%). Huai’an and Yangzhou are two areas with high ACED because these areas are important food commodity bases in China, and rice is their main crop, and the annual planting area accounts for more than 60% of the regional crop sowing area. Currently, crop fertilization practices in these areas are relatively crude, leading to high ACED. Huai’an’s crop area rose from 9.74% of the province in 2005 to 10.83% in 2020, but its fertilizer application ratio rose from 9.61% to 11.82%. Yangzhou’s crop area rose from 9.74% of the province in 2005 to 6.39% in 2020, but its fertilizer application rate rose from 5.34% to 6.43%. This means that fertilizer use in these two cities is growing faster than crop area growth. The largest decrease in ACED in Wuxi is mainly due to the decrease in its share of the food crop area, from 64.14% of the province in 2005 to 54.21% in 2020.

### 3.2. Global Spatial Autocorrelation Analysis of ACEs

#### 3.2.1. Global Autocorrelation Analysis

The global Moran’s I index of ACEs and ACED in Jiangsu Province from 2005 to 2020 was measured by using the spatial analysis software GeoDa 1.14 and selecting the binary adjacency matrix, and the calculated results are shown in Table 5.

The global Moran’s I index of ACEs for all cities in Jiangsu Province from 2005 to 2020 is positive, indicating that there is a global autocorrelation of ACEs in Jiangsu Province, i.e., ACEs as a whole tend to influence and interact with each other, and neighboring regions have similar characteristics of ACEs due to their geographical proximity and the joint effect of other factors. Among them, the global Moran’s index of ACEs lies between 0.215 and 0.483, with a mean value of 0.394, and the *p*-values all pass the significance test at the 5% level, showing a relatively smooth upward trend, and the Moran’s I value reaches its maximum in 2019, with the greatest spatial correlation. The global Moran’s I index of ACED is overall lower than that of ACEs, with a mean value of 0.024, and does not pass the significance test, indicating that the spatial distribution of ACED is characterized by randomness.

**Figure 6:**
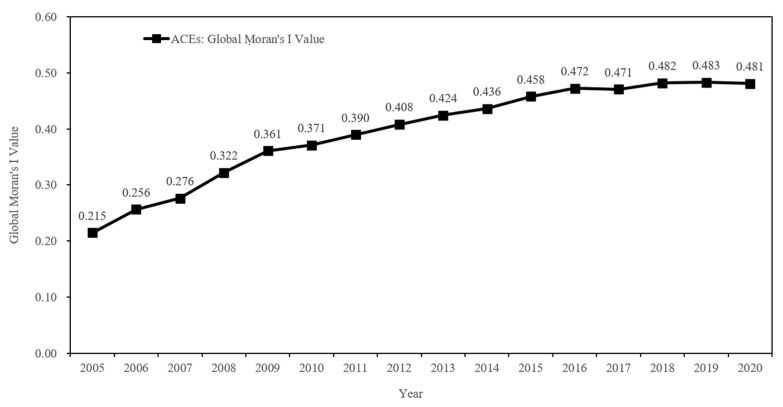
Trend of Moran’s I index values for ACEs in Jiangsu Province from 2005 to 2020.

The trend of the global Moran′s I index values shows that the Moran′s I values for ACEs in Jiangsu Province show an increasing trend during the study period (Figure 6). This indicates that the spatial clustering of ACEs has been strengthened with the evolution of time.

#### 3.2.2. Local Autocorrelation Analysis

**Figure 7:**
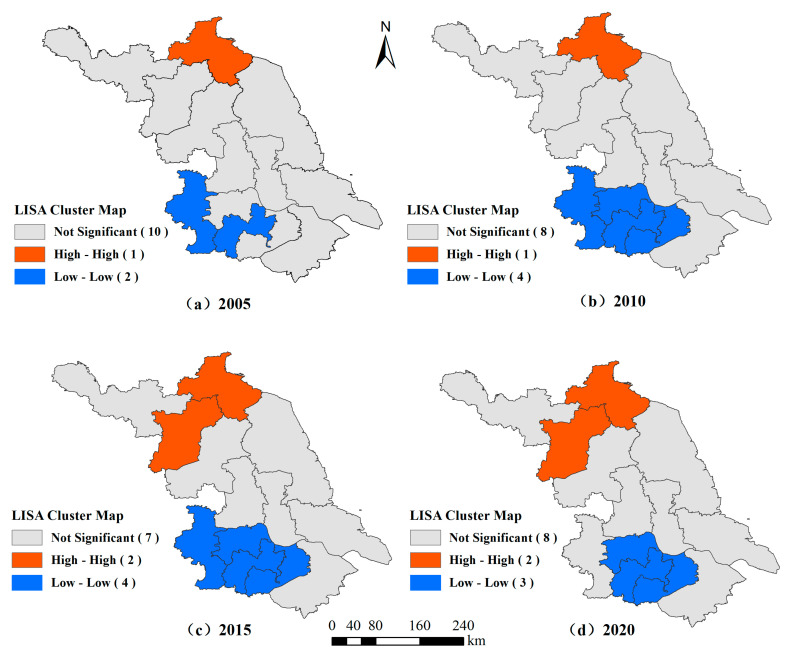
Local spatial autocorrelation LISA aggregation of ACEs in Jiangsu Province in Main Years.

To present the spatial clustering characteristics of ACEs in Jiangsu Province more visually, LISA clustering maps were drawn for 2005, 2010, 2015, and 2020 (Figure 7).

The ACEs from 13 cities in Jiangsu Province show a more obvious divergence pattern, and the cities with high-emission clusters are mainly Lianyungang and Suqian, i.e., the levels of ACEs in these cities themselves and the neighboring cities are higher. The main reason is that these cities have flat topography, sufficient water and heat, rain and heat in the same period, and good agricultural bases, which are the main commercial food bases in Jiangsu. The cities with obvious low-emission clusters in the region are mainly Zhenjiang, Changzhou, and Wuxi, i.e., these cities have fewer ACEs themselves and the surrounding cities, mainly because these cities have a higher level of economic development and have a greater advantage in developing industry, so their ACEs are not high. The sum of cities located in the H-H agglomeration and L-L agglomeration accounts for 38.46% of all cities in Jiangsu, indicating that the spatial spillover effect of ACEs still needs to be strengthened. Since 2005, the number of cities with high and low emission concentrations has been increasing, but the overall number of cities with low emission concentrations are still the majority, which indicates that the situation of green agricultural development in Jiangsu Province is improving.

### 3.3. Analysis of Driving Factors of ACEs

#### 3.3.1. STIRPAT Model Process

To avoid the effect of multicollinearity among variables, factor analysis is used to extract common factors for regression analysis of the seven variables [67]. First, after dimensionless processing of the raw data, the KMO and Bartlett’s tests are performed on the seven indicators using SPSS 20.0 software, and the results showed that the value of KMO was 0.735 (a KMO value > 0.7 indicates suitability for factor analysis) and the *p*-value was 0.000 (*p* < 0.05 indicates the existence of a correlation between indicators) [68], indicating the existence of a correlation between indicators and suitability for factor analysis.

Then, a principal component analysis is performed on the indicators with the criteria of eigenvalues greater than one and a cumulative variance contribution rate not less than 80% [69], and it was found that the former two components summarized 98.735% of the information content of the original variables (Table 6), so the former two components are extracted as common factors, and the factor loading matrix is further rotated using the maximum variance orthogonal rotation method to obtain the factor score coefficient status (Table 7).

Subsequently, the expressions between the two common factors and the seven variables were obtained from the factor score coefficient matrix.

(10) $$FAC_{1}=-0.176\mathrm{In}P+0.197\mathrm{In}A+0.182\mathrm{In}T-0.263\mathrm{In}V+0.174\mathrm{In}U+0.179\mathrm{In}C+0.192\mathrm{In}R\;\;\;\;$$

(11) $$FAC_{2}=0.023\mathrm{In}P-0.089\mathrm{In}A-0.038\mathrm{In}T+1.088\mathrm{In}V-0.011\mathrm{In}U-0.031\mathrm{In}C-0.069\mathrm{In}R\;\;\;\;\;\;$$

Then, based on factor analysis, multiple regression analysis is performed between the two common factors and lnCE (Table 8). The regression results showed that the R^2^ of the model is 0.546, the *p*-value is 0.000, and the coefficients of all variables are less than 0.05, indicating that the equations are well fitted, resulting in the regression equations of lnCE with *FAC*_1_ and *FAC_2_* as follows.

(12) $$InCE=-0.632FAC_{1}+0.024FAC_{2}\;\;\;\;$$

Finally, substituting Equations (10) and (11) into Equation (12) yields.

(13) InCE=0.112InP−0.127InA−0.116InT+0.192InV−0.110InU−0.114InC−0.123InR  

This leads to the STIRPAT model of ACEs in Jiangsu Province.

(14) $$CE=P^{0.112}A^{-0.127}T^{-0.116}V^{0.192}U^{-0.110}C^{-0.114}R^{-0.123}\;\;$$

#### 3.3.2. Analysis of STIRPAT Results

From Equation (14), it can be seen that each 1% change in the rural population, economic development level, agricultural technology factors, agricultural industry structure, urbanization level, rural investment, and per capita disposable income of farmers causes changes of 0.112%, −0.127%, −0.116%, 0.192%, −0.110%, −0.114%, and −0.123% in Jiangsu’s ACEs, respectively. Among them, two factors, namely, rural population and agricultural industry structure, play a role in promoting agricultural carbon emissions in Jiangsu Province, among which the agricultural industry structure plays the largest role in promoting carbon emissions, followed by the rural population. The level of economic development, agricultural technology factors, urbanization level, rural investment, and farmers’ per capita disposable income play a role in suppressing carbon emissions, among which the level of economic development has the most obvious suppression effect, followed by rural residents’ per capita disposable income and agricultural technology level, and the urbanization rate has the weakest suppression effect.

The rural population and the structure of the agricultural industry show a positive effect on ACEs. This is because the topography of Jiangsu Province is dominated by plains, and planting dominates the agricultural sector. The increase in the rural population leads to an increase in the number of people engaged in planting production, which indirectly promotes the use of agricultural chemicals such as chemical fertilizers. The increase in urbanization rate means that the rural population is transformed into an urban population, the proportion of tertiary industry in the economic structure increases, and the proportion of primary and secondary industries decreases, while the increase in agricultural informatization, the change of farmers’ lifestyle, and the establishment of modern agricultural management systems all contribute to the reduction in ACEs.

The level of agricultural economic development and the per capita disposable income of rural residents are the main inhibiting factors driving the growth of ACEs. According to the environmental Kuznets theory, economic development and environmental conditions will show an inverted “U” curve, that is, the environmental conditions will go through a process of deterioration and then treatment with economic development. At present, the ACEs in Jiangsu Province are at the "inflection point" of the environmental curve, and the ACEs show an obvious downward trend with the development of the agricultural economy. As the size of the economy grows and the disposable income of rural residents increases, residents’ preferences will shift from consumption of low-grade to high-grade goods. They are now more willing to purchase green agricultural products produced according to stricter environmental standards. Therefore, the higher income of rural residents helps establish stricter environmental and taxation standards, reducing the intensity of agricultural emissions per unit of output.

The degree of agricultural mechanization has an abatement effect on ACEs. On the one hand, the development of agricultural mechanization enhances farming efficiency, promotes the withdrawal of small farmers from agricultural production, inhibits the excessive application of fertilizers caused by farmers’ single pursuit of yield, and allows conservation farming practices to be applied on a larger scale. On the other hand, agricultural mechanization helps to rationalize and optimize the agricultural cultivation structure and reduce the intensity of ACEs by expanding the cultivation area of food crops with relatively low-carbon emissions from crop production. Investment in agricultural fixed assets has a suppressive effect on ACEs, but the extent of its influence is low compared with several other factors. Investment in agricultural fixed assets mainly consists of productive biological assets and public welfare biological assets, for example, expenditures on the acquisition of economic forests, expenditures on the construction of wind and sand control forests, soil and water conservation forests, and water connotation forests, which are production or reproduction activities that absorb part of the greenhouse gases and result in a reduction of CO_2_ emissions.

## 4. Discussion

Based on 10 types of carbon sources, including agricultural land use, rice cultivation, and livestock and poultry breeding, this study conducted in-depth research on the spatial and temporal distribution characteristics and influencing factors on ACEs in Jiangsu Province, so as to grasp the current situation of ACEs in Jiangsu Province as a whole, which can provide practical guidance for Jiangsu Province to accelerate the implementation of agricultural transformation and upgrades and high-quality agricultural development. The research results show that in the past 16 years, Jiangsu Province has also made certain achievements in ACEs reduction, as shown in the total decrease in ACEs, from 1877.57 × 10^4^ t in 2005 to 1795.24 × 10^4^ t in 2020, with an average annual decrease of 0.32%. However, some problems have also been identified, such as large differences in the total amount and ACED between regions. In future work on ACEs reduction, on the one hand, we should ensure the sustainable growth of the agricultural economy and hold the bottom line of food security, and on the other hand, we should develop differentiated carbon reduction policies according to urban characteristics.

The implications of our policy are as follows:

(1) Government departments should make energy saving and emission reduction policies according to local conditions, and the implementation of carbon emission reduction should focus on the areas with high ACEs and prevent the expansion of regional differences in ACEs. For high ACEs areas, to transform into low-carbon agriculture, these areas should scientifically plan the layout of agricultural industries, accelerate the pace of low-carbon science and technology innovation in agriculture, and appropriately reduce the use of agricultural materials such as pesticides, chemical fertilizers, and agricultural films. For low ACEs areas to continue to optimize the structure of agricultural production, they should vigorously develop leisure agriculture, ecological agriculture, and urban agriculture with higher agricultural output values, etc., so that they can move in the direction of less carbon emission development.
(2) Urbanization has a suppressive effect on ACEs, and the construction of new urbanization should be promoted, especially the urbanization of the population, to realize the optimization of industrial structure and the development of clean production through the aggregation and scale of population, land, and other resource factors, thus promoting the smooth transfer of surplus rural labor.
(3) Under the premise of ensuring food security, the industrial structure of plantation and forestry, animal husbandry, and fishery industries need to be further optimized to achieve coordinated development of agriculture, forestry, animal husbandry, and fishery industries. Of course, in the process of agricultural modernization, the promotion of agricultural mechanization should focus on the use of efficiency and strive to reduce the waste of ineffective resources.

In our future research, in terms of content, we will try to incorporate straw burning, runoff, and leaching into the ACEs measurement index system, deeply explore the reasons for the differences in regional ACEs, and focus on the relationship between agricultural economic growth and ACEs reduction. In terms of methodology, we will further learn machine learning algorithms such as random forest and try to use them as our research tools.

## 5. Conclusions

This paper measures and analyzes the spatial and temporal characteristics of ACEs in Jiangsu Province from 2005 to 2020, explores the spatial clustering characteristics of ACEs using the global spatial autocorrelation method, and explores the factors influencing ACEs using the STIRPAT model, with the following main findings:

(1) Jiangsu’s ACEs decreased from 1877.57 × 10^4^ tons in 2005 to 1795.24 × 10^4^ tons in 2020, with an average annual decrease of 0.32%, while the ACED increased from 240.06 t/km^2^ in 2005 to 245.72 t/km^2^ in 2020, with an increase of 2.36%. In terms of stages, the trend of “rapid growth—slow decline—accelerated decline” is more obvious; in terms of regions, the high ACEs areas are concentrated in the northern Jiangsu region.
(2) The global Moran’s I index of total ACEs in Jiangsu Province from 2005 to 2020 is positive, ranging from 0.215 to 0.483, with a mean value of 0.394, and the spatial agglomeration is increasing, and the spatial distribution of ACED shows a random characteristic. The local spatial autocorrelation analysis shows that the ACEs in Jiangsu Province form a high–high emission agglomeration centered on Lianyungang and Suqian and a low–low emission agglomeration centered on Zhenjiang, Changzhou, and Wuxi.
(3) Each 1% change in the rural population, economic development level, agricultural technology factor, agricultural industry structure, urbanization level, rural investment, and disposable income per farmer causes changes of 0.112%, −0.127%, −0.116%, 0.192%, −0.110%, −0.114%, and −0.123% in Jiangsu’s ACEs, respectively. Among them, the two factors, rural population and agricultural industry structure, play a role in promoting ACEs; the level of economic development, agricultural technology factors, urbanization level, rural investment, and per capita disposable income of farmers play a suppressive role. The agricultural industry structure has the greatest role in promoting ACEs, and the level of economic development has the most obvious suppressive effect.

## Figures and Tables

**Table 1:** Descriptive statistics of the main variables.

Variable	Mean	SD	Min	Max
ACEs/(10^4^t)	1868.17	43.08	1766.69	1913.94
ACED/(t/km^2^)	246.93	5.35	237.38	256.34
Urbanization rate/(%)	62.81	7.72	50.50	73.40
Rural population/(10^4^)	2988.41	502.49	2251.40	3756.18
Total agricultural output/(10^8^ CNY)	5470.46	1866.34	2576.98	7952.59
Disposable income per rural resident/(CNY)	13,376.75	6243.85	5258.00	24,198.00
Total power of agricultural machinery/(10^4^ kw)	4290.88	701.84	3135.33	5214.83
Total fixed asset investment in agriculture/(10^8^ CNY)	311.81	198.52	54.79	608.99
Share of output value of farming and animal husbandry/(%)	71.19	2.47	67.10	74.22

**Table 2:** ACEs sources and corresponding carbon emission coefficients.

Carbon Category	Carbon Source	Coefficient	Unit	Refer Source
Agricultural land utilization	Pesticide	4.934	Kg C/Kg	ORN
	Agricultural plastic films	5.180	Kg C/Kg	IREEA
	Fertilizer	0.896	Kg C/Kg	OPNL
	Agricultural diesel oil	0.593	Kg C/Kg	IPCC (2007)
	Agricultural irrigation	266.480	Kg C/Hm^2^	Duan et al. [50]
	Agricultural cultivation	312.600	Kg C/Km^2^	Wu et al. [51]
Rice cultivation	Rice	5110.92	Kg C/Hm^2^	Liu et al. [52]
Livestock breeding emissions	Cattle	415.91	Kg C/Year	IPCC (2007)
	Sheep	35.182	Kg C/Year	IPCC (2007)
	Pigs	34.091	Kg C/Year	IPCC (2007)

Note: ORNL is Oak Ridge National Laboratory; IPCC is Intergovernmental Panel on Climate Change; IREEA is Institute of Agricultural Resources, Ecosystems and Environment, Nanjing Agricultural University.

**Table 3:** ACEs in Jiangsu Province, China from 2005 to 2020.

Year	Agricultural Land Utilization		Rice Cultivation		Livestock and Poultry Breeding		ACE /10^4^ t	Growth Rate /%	ACED (t/km^2^)
	CE /10^4^ t	PERC /%	CE /10^4^ t	PERC /%	CE /10^4^ t	PERC /%			
2005	548.45	29.21	1210.76	64.49	118.36	6.30	1877.57	-	240.06
2006	557.97	29.47	1224.50	64.68	110.68	5.85	1893.15	0.83	237.38
2007	557.45	29.61	1224.47	65.03	100.88	5.36	1882.80	−0.55	241.38
2008	559.82	29.83	1220.78	65.05	96.19	5.13	1876.80	−0.32	242.17
2009	566.29	29.96	1219.13	64.50	104.80	5.54	1890.23	0.72	244.07
2010	569.60	29.93	1221.50	64.17	112.34	5.90	1903.44	0.70	243.44
2011	566.50	29.60	1223.32	63.92	124.12	6.49	1913.94	0.55	247.25
2012	563.23	29.62	1225.63	64.46	112.45	5.91	1901.31	−0.66	247.69
2013	560.10	29.52	1224.89	64.55	112.54	5.93	1897.52	−0.20	248.49
2014	559.31	29.46	1227.02	64.63	112.23	5.91	1898.57	0.06	250.31
2015	549.84	29.19	1223.61	64.96	110.20	5.85	1883.64	−0.79	249.89
2016	543.69	29.16	1220.42	65.45	100.56	5.39	1864.67	−1.01	250.61
2017	534.01	29.18	1203.75	65.78	92.20	5.04	1829.95	−1.86	250.41
2018	522.04	28.76	1211.59	66.75	81.62	4.50	1815.25	−0.80	255.72
2019	515.19	29.16	1203.42	68.12	48.08	2.72	1766.69	−2.68	256.34
2020	508.73	28.34	1210.19	67.41	76.32	4.25	1795.24	1.62	245.72
AAGR/%	−0.50	−	0.00	−	−2.88	−	−0.30		0.16

**Table 4:** Changes in ACEs and ACED in Main Years.

City	2005		2010		2015		2020	
	ACEs (10^4^ t)	ACED (t/km^2^)	ACEs (10^4^ t)	ACED (t/km^2^)	ACEs (10^4^ t)	ACED (t/km^2^)	ACEs (10^4^ t)	ACED (t/km^2^)
Nanjing	96.87	241.42	79.53	237.21	76.91	242.70	66.41	264.09
Wuxi	63.46	339.34	51.54	284.90	39.87	230.28	31.52	242.86
Xuzhou	204.74	199.80	215.39	195.97	211.89	182.56	189.85	160.83
Changzhou	68.77	285.49	62.36	269.92	57.48	267.79	42.45	257.19
Suzhou	74.53	239.79	71.64	265.43	63.87	255.27	54.93	262.01
Nantong	164.71	188.72	158.63	185.54	152.15	182.05	146.41	186.20
Lianyungang	150.06	270.43	163.77	276.69	166.89	263.34	166.69	263.33
Huai’an	266.37	358.62	282.81	362.79	290.69	365.34	299.56	369.92
Yancheng	270.85	198.78	297.66	203.86	303.53	212.73	307.06	221.02
Zhenjiang	68.03	291.41	62.99	264.34	60.73	257.34	49.55	273.81
Taizhou	148.38	264.54	142.99	249.99	140.34	241.54	129.18	249.10
Suqian	159.37	238.62	170.08	241.60	173.58	243.80	174.94	234.40
Yangzhou	141.44	301.80	144.05	288.02	145.72	286.21	136.69	286.06

**Table 5:** Moran’s I value and *p*-value for ACEs and ACED in Jiangsu.

Year	ACEs	Z-Value	*p*-Value	ACED	Z-Value	*p*-Value
2005	0.215	1.871	0.049	0.023	0.579	0.256
2006	0.256	2.105	0.033	−0.010	0.411	0.305
2007	0.276	2.216	0.025	−0.015	0.408	0.323
2008	0.322	2.448	0.022	−0.006	0.446	0.302
2009	0.361	2.673	0.012	−0.022	0.362	0.335
2010	0.371	2.720	0.010	−0.026	0.344	0.354
2011	0.390	2.847	0.012	−0.008	0.462	0.318
2012	0.408	2.912	0.009	−0.014	0.433	0.336
2013	0.424	3.044	0.007	−0.002	0.496	0.307
2014	0.436	3.066	0.005	0.008	0.571	0.283
2015	0.458	3.104	0.004	0.009	0.589	0.276
2016	0.472	3.254	0.004	0.027	0.677	0.267
2017	0.471	3.245	0.003	0.059	0.888	0.195
2018	0.482	3.270	0.005	0.045	0.793	0.228
2019	0.483	3.248	0.003	0.015	0.593	0.281
2020	0.481	3.149	0.004	0.308	0.683	0.249

**Table 6:** Principal component analysis results of variables.

Factors	Initial Eigenvalues		Extraction Sums of Squared Loadings			
	Total	% of Variance	Cumulative %	Total	% of Variance	Cumulative %
1	6.146	87.805	87.805	6.146	87.805	87.805
2	0.765	10.930	98.735	0.765	10.930	98.735
3	0.056	0.802	99.537			
4	0.021	0.299	99.837			
5	0.009	0.132	99.969			
6	0.001	0.020	99.989			
7	0.001	0.011	100.000			

**Table 7:** Score coefficient matrix of principal component analysis.

Variable	Factors	
	FAC_1_	FAC_2_
InP	−0.176	0.023
InA	0.197	−0.089
InT	0.182	−0.038
InV	−0.263	1.088
InU	0.174	−0.011
InC	0.179	−0.031
InR	0.192	−0.069

**Table 8:** OLS regression results of principal components.

Parameter	Unnormalized Coefficient		Standardization Coefficient	*t*-Test	*p*-Value
	Nonstandard Coefficient	SE	Beta		
Constant	0.000	0.208	0.000	0.000	1.000
FAC_1_	−0.632	0.215	−0.632	−2.944	0.011
FAC_2_	0.024	0.215	0.024	0.113	0.412

## Data Availability

Data will be available if necessary.

## Appendix A

Jiangsu Province is a provincial administrative region of the People’s Republic of China. Nanjing, the capital of the province, is located in the Yangtze River Delta region, on the eastern coast of mainland China, spanning latitude 30°45′~35°08′ N and longitude 116°21′~121°56′ E. It shares borders with Shanghai, Zhejiang, Anhui, and Shandong provinces.

Jiangsu Province can be divided into three major regions: Northern Jiangsu, Central Jiangsu, and Southern Jiangsu

Central Jiangsu Province: Nantong, Yangzhou, and Taizhou

Southern Jiangsu Province: Nanjing, Suzhou, Wuxi, Changzhou, and Zhenjiang

Northern Jiangsu: Xuzhou, Lianyungang, Suqian, Huai’an, and Yancheng
